# Thirty Cases of Primary Suture for Complicated Fractures

**Published:** 1918-03

**Authors:** 


***Thirty Cases of Primary Suture for Complicated Fractures.*** By M. GASTON PICOT, Médecin aide-major, Reported by PIERRE DUVAL. Trans. and Abs. from the *Bulletins et Mémoires de la Société de Chirurgie de Paris*, Oct. 16, 1917.

Thirty cases of diaphyseal fracture, treated by primary suture, are presented and discussed. The bacteriological and surgical technique are described, and statistics from these and other cases are given.

P. removes sub-periosteal splinters freely, respecting only the continuity of the bone. All bone surfaces are treated with minute care. All fracture surfaces are thoroughly curetted, and even the medullary canal is curetted for a distance of about 2 cm. If there are any long fissures, they are opened slightly with a wedge and curetted upon both surfaces. The use of Lambotte clamps and the bending of the limb at the point of the fracture greatly assist in the work of cleansing. All the bone surfaces are then rubbed with a swab saturated with ether. This application should be several times repeated. Small splinters should be treated with as much care as the more important ones.

The surfaces to be sutured are then brought together, and capillary drains inserted to the depth of the bone. The skin should be brought together as perfectly as possible.

The bacteriological control is carried out by means of a pipette inserted along the tract of the drains. Tissier's rules[1] are carefully observed.

Out of 30 cases thus treated, P. reports 25 perfect unions, 3 fistulous complications, and 2 failures.

Bacteriological tests showed 13 cases to be completely aseptic, and 17 of the cases to be contaminated only with the less dangerous organisms mentioned by Tissier. The organisms present in one of the two cases that failed were Coli communis and staphylococcus, and in the other, staphylococcus and anaerobes.

D. reports that Picot's technique was used by the surgeons of his Ambulance with only slightly inferior results. Out of 35 fractures treated by immediate or slightly delayed suture, 20 succeeded perfectly. In 5 cases the skin gave way, but granulations had formed over the bone. In only 1 case was the operation a complete failure.

The authors insist upon the great importance of complete fixation from the earliest possible moment. They consider the Thomas splint by far the most satisfactory appliance for this purpose. They also used the Blake and Hodgens suspension methods, with or without the Finochietto stirrup.

1. See *The Medical Bulletin*, No 1, for Nov. 1917, p. 33 for an abstract of Tissier's article in the *Bull. et Mém. de la Soc. de Chir.*, July 10, 1917.

